# Dysregulation of Ribosome Biogenesis and Translational Capacity Is Associated with Tumor Progression of Human Breast Cancer Cells

**DOI:** 10.1371/journal.pone.0007147

**Published:** 2009-09-25

**Authors:** Stéphane Belin, Anne Beghin, Eduardo Solano-Gonzàlez, Laurent Bezin, Stéphanie Brunet-Manquat, Julien Textoris, Anne-Catherine Prats, Hichem C. Mertani, Charles Dumontet, Jean-Jacques Diaz

**Affiliations:** 1 Inserm, U590, Lyon, France; 2 Université Lyon 1, ISPB, Lyon, France; 3 Université de Lyon, Université Lyon 1, CNRS, UMR 5534, Centre Léon Bérard, Lyon, France; 4 Université de Lyon, Université Lyon 1, CNRS, UMR 5123, Centre Léon Bérard, Lyon, France; 5 Inserm, U858, Toulouse, France; 6 Université de Toulouse, UPS, Institut de Médecine Moléculaire de Rangueil, IFR150, Toulouse, France; Victor Chang Cardiac Research Institute (VCCRI), Australia

## Abstract

Protein synthesis is a fundamental cell process and ribosomes - particularly through the ribosomal RNA that display ribozyme activity - are the main effectors of this process. Ribosome biogenesis is a very complex process involving transcriptional as

well as many post-transcriptional steps to produce functional ribosomes. It is now well demonstrated that ribosome production is enhanced in cancer cells and that ribosome biogenesis plays a crucial role in tumor progression. However, at present there is an important lack of data to determine whether the entire process of ribosome biogenesis and ribosome assembly is modified during tumor progression and what could be the potential impact on the dysregulation of translational control that is observed in cancer cells. In breast cancer cells displaying enhanced aggressivity, both *in vitro* and *in vivo*, we have analyzed the major steps of ribosome biogenesis and the translational capacity of the resulting ribosome. We show that increased tumorigenicity was associated with modifications of nucleolar morphology and profound quantitative and qualitative alterations in ribosomal biogenesis and function. Specifically cells with enhanced tumor aggressivity displayed increased synthesis of 45S pre-rRNA, with activation of an alternative preRNA synthetic pathway containing a 43S precursor and enhanced post-transcriptional methylation of specifc sites located in the 28S rRNA. While the global translational activity was not modified, IRES-initiated translation, notably that of p53 mRNA, was less efficient and the control of translational fidelity was importantly reduced in cells with increased aggressivity. These results suggest that acquisition of enhanced tumor aggressivity can be associated with profound qualitative alterations in ribosomal control, leading to reduced quality control of translation in cancer cells

## Introduction

Nucleoli were among the first structures of eucaryotic cell nuclei to have been identified and functionally characterized. Nucleoli are now recognized as multifunctional domains containing some 700 different types of proteins involved in several fundamental processes of cell life such as ribosome biogenesis, RNA maturation, cell cycle and apoptosis regulation [1]–[3]. It has been known for many years that nucleoli undergo to profound morphological alterations in cancer cells. Hypertrophy of the nucleolus, visualized by analysis of silver-stained nucleolar organizer regions (AgNORs) is one of the most distinctive cytological features of cancer cells [4], [5]. AgNOR analysis is even considered as a “gold standard” when retrospective studies on archival, formalin-fixed, and paraffin-embedded material are performed [6]. AgNOR analysis is an independent prognostic factor for almost every cancer analyzed so far and is regarded as one of the major and independent prognostic factor for clinical decision making in colorectal adenocarcinoma as well as invasive prostate cancer [5], [6].

Ribosome biogenesis takes place mainly within nucleoli and is characterized by the ultra-structural organization in three main compartments, the fibrillar centers (FC) containing rDNA gene clusters, the dense fibrillar centers (DFC) where rDNA gene transcription and post-transcriptional processing occurs and the granular component (GC) in which final maturation and assembly takes place [7]. Ribosomal biogenesis involves probably more than 170 accessory factors [2] and requires transcriptional and post-transcriptional steps synchronized in a timely and spatially regulated [8]. Eukaryotic ribosomes are constituted of four different ribosomal RNAs (rRNA) synthetized by RNA polymerase I (RNA pol I) and RNA polymerase III (RNA pol III) that are assembled with eighty different ribosomal proteins to form the 40S and 60S subunits. In mammals, the 40S subunit contains the 18S rRNA whereas the 60S subunit contains the 28S, 5.8S and 5S rRNA. These rRNA are first synthesized as precursor rRNA (pre-rRNA) molecules and undergo extensive co- and post-transcriptional processing to produce completely matured and functional rRNA. This processing includes specific endo- and exo-nucleotidic cleavages to remove all the transcribed sequences from the pre-rRNA that are not part of the matured rRNA [9], [10] as well as specific methylation and pseudouridylation induced by ribonucleoproteins composed of small nucleolar RNA (snoRNA) of two types (snoRNA C/D and snoRNA H/ACA) associated with either fibrillarin for the methylation or dyskerin for pseudouridylation [11]–[13]. The precise function of these chemical modifications is not yet fully elucidated but several recent data strongly suggest that they modulate ribosomal assembly and translational capacity [14]–[19]. Experimental data are however still missing to determine the regulations of ribosome biogenesis and in particular those affecting the post transcriptional steps during human carcinoma cell progression, although recent studies indicate that these alterations might directly contribute to tumorigenesis [18]

Oncogenesis and tumor progression in humans are supported by the acquisition of activated oncogenes and/or the silencing of tumor suppressor [20]. Recent data have associated the activity of certain oncogenes and tumor suppressor with precise regulation of ribosome processing [21]–[23]. For example, oncogenes such as c-myc activate not only RNA pol II (involved in synthesis of mRNA coding for ribosomal proteins) but also RNA pol I and III (involved in rRNA synthesis) [23]. Moreover, the loss of functional changes in the two major tumor suppressor proteins pRB and TP53 activities induced a strong activation of ribosome biogenesis in cancer cell [24]. More importantly, it has been shown that oncogenic activity of c-myc could be suppressed by the direct genetic control of ribosome biogenesis through a process that involved the restoration of the IRES-dependent translational ability of the cell that is otherwise deregulated by c-myc [25].

We have recently found that reduced content of ADP ribosylation factor like 2 (Arl2) in breast tumor lines is associated with enhanced *in vitro* and *in vivo* aggressivity (Beghin et al., submitted for publication). Analysis of variant lines of the human breast adenocarcinoma lines MCF7 and MDA-MB-231 showed that cells with reduced Arl2 content displayed reduced contact inhibition, increased clonogenic or cluster formation as well as a proliferative advantage over control cells in an *in vitro* competition assay. These cells also caused larger tumors in SCID mice, a phenotype which was mimicked by the *in vivo* administration of siRNA directed against Arl2 or PP2A.

In the present study we have analyzed the rates and quality of ribosome biogenesis and rRNA processing as well as the translational capacity in cells with reduced Arl2 content [26], [27]. We show that the acquisition of an aggressive phenotype in these cells is accompanied by a strong activation of ribosome biogenesis supported by i) activation of the rate of ribosome synthesis, ii) alteration of the rRNA processing pathways and of their methylation pattern; iii) impairment of the translational initiation ability via CAP or IRES-dependent mechanisms and iv) an important alterations of translational fidelity. These results show that the acquisition of an aggressive tumor phenotype is associated with profound quantitative and qualitative alterations in ribosomal biogenesis and function.

## Results

### Reduced Arl2 content is associated with profound alterations in nucleolar morphology

**Figure 1:**
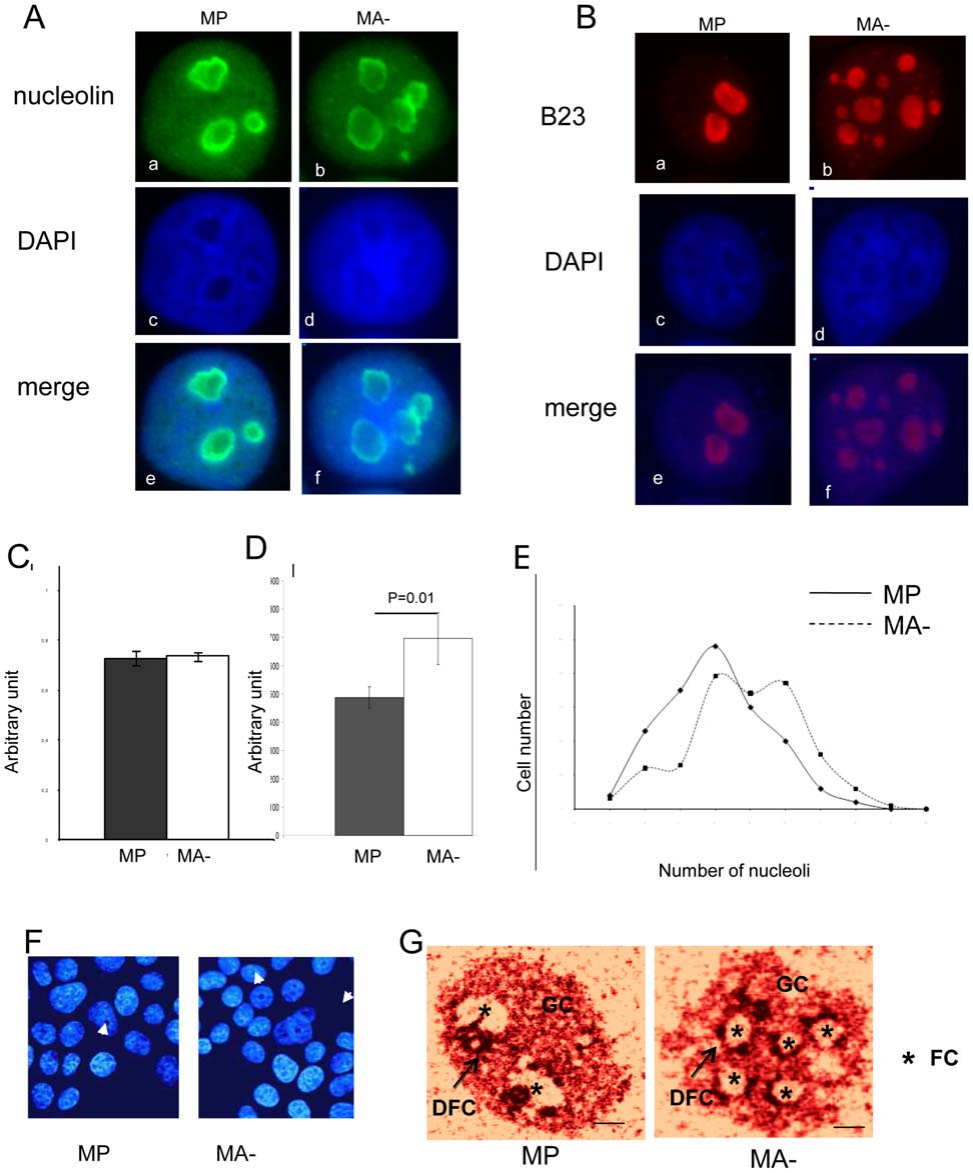
The nucleolar structure is profoundly altered in aggressive human breast cancer cells. Immunofluorescent detection of the nucleoli of the two cell lines using the nucleolin antibody (A) and the B23 antibody (B) on panels a and b. The nuclei are identified using DAPI staining (blue signal) on panels c and d and images of merged signals are presented on panels e and f. (C) Analysis of the circularity of the nucleoli was performed as described in material and methods using Image J software, and no significant differences were observed between the two cell lines. The same image analysis was conducted for the calculation of the nucleolar area (D), demonstrating that nucleoli of MA- cells exhibit nucleoli 30% larger than those of MP cells (p = 0.01). The number of nucleoli per nucleus was quantified in MP and MA- cell (E). The graphic shows that MP cells have an average of 4 nucleoli per cell whereas the average for MA- cells is about 6 nucleoli per cell (p = 0.01). (F) Confocal microscopic analysis after DAPI staining showed that 40% of MA- cells *vs*. MP cells present very dense nucleoli (p≤0.01). Electron microscopic analysis of MA- and MP nucleoli is presented in (G). The nucleoli of MA- cells exhibit significantly more fibrillar center as compared to those of MP cells (p≤0.01). GC: granular component, DFC: dense fibrillar component, FC: fibrillar center represented by a star. Scale bar is 1,2 µm.

Since the size, the morphology, the number and the ultra-structural organisation of nucleoli are considered as potent indicators of cellular nucleolar activity, the first part of our study aimed to determine these characteristics in the more aggressive MA- cells and the control MP cells. Nucleoli were visualized by immunofluorescence experiments using antibodies directed against two abundant nucleolar proteins, nucleolin and B23. Detection of nucleolin and B23 signal is presented in figure 1A and 1B panel a and b, the nucleolus is revealed by DAPI staining (panel c and d) and the merged picture is presented in panel e and f. The morphology and the number of nucleoli is very different in the most aggressive cells compared with the MP control cells. It was confirmed by signal detection of these staining by software analysis. The circularity index (Fig. 1C) and the area (Fig. 1D) of the nucleoli were then evaluated from 160 cells of each cell line using the Image J software. These analyses showed that there was no statistically significative differences between circularity index of nucleoli from MP or MA- cells whereas the mean area of nucleoli from MA- cells was 30% higher than that of nucleoli from MP cells (p = 0.01). Finally, quantification of the number of nucleoli per cell (Fig. 1E) showed that the number of nucleoli varies from 1 up to 10 per cell in both cell lines but invasive mammary carcinoma cells MA- exhibit a higher proportion of nuclei containing more than 4 nucleoli as compared to the MP control cell (p = 0.01, Chi2 test). Confocal microscopy following DAPI staining showed that approximately 40% of MA- cells displayed very dense nucleoli as quantified into 200 cells using the ImageJ software (p≤0.01)(Fig. 1F). Electron microscopy showed that the number of FC and DFC per nucleoli is higher in MA- cells compared to MP cells, aproxymatively 4 FC per nucleolus in MA- cells (p≤0.01, t-test). A representative image is presented in Fig. 1G. Altogether these results suggested that during human breast cancer progression the nucleus undergo adaptative changes supported by increased nucleolar activity.

### Reduced Arl2 content is associated with increased ribosome biogenesis

**Figure 2:**
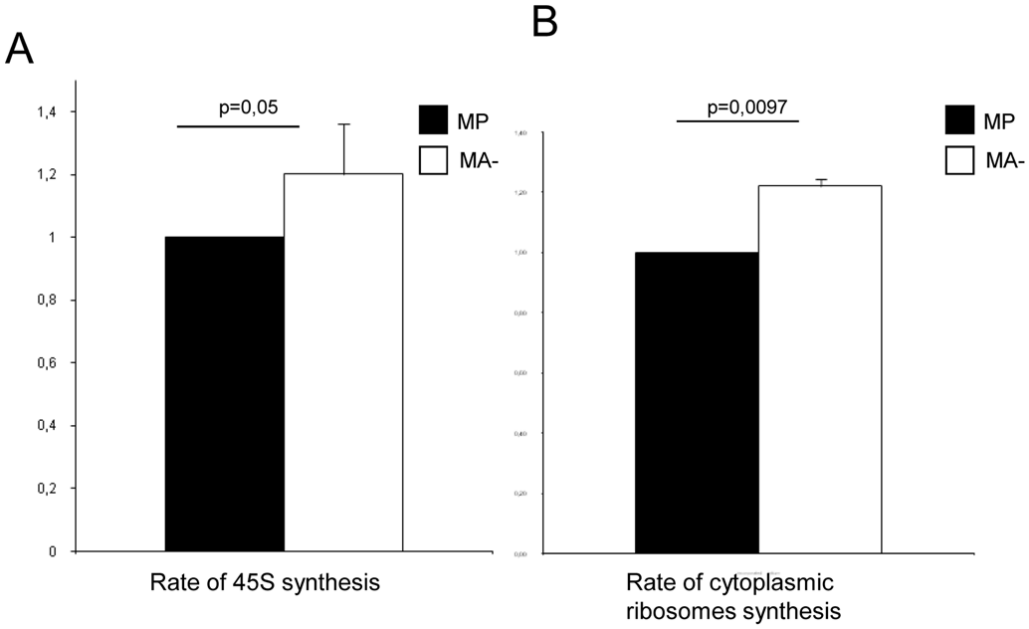
Polymerase I activity and ribosome biogenesis are activated in aggressive human breast cancer cells. A: RNA polymerase I activity. The activity of the RNA *pol* I was investigated in MP and MA- cells by counting the radioactivity directly incorporated into the 45S pre-rRNA . Quantification of the amount of [^3^H]-uridine incorporated into pre-rRNA 45S show a 20% increase in MA- cells (p = 0.05, n = 3). The amount of radioactivity is expressed as percentage with the result obtained for MP cells taken as the reference and is presented as mean +− S. E. M (n = 3). B: Rate of cytoplasmic ribosome biogenesis. The rate of ribosome biogenesis was evaluated by counting the radioactivity incorporated into purified mature cytoplasmic ribosome. Quantification of the amount of [^3^H]-uridine incorporated into cytoplasmic ribosome (determined as above) demonstrate a 20% increase in cytoplasmic ribosome biogenesis in MA- cells as compared to control MP cell (n = 3).

The rate of ribosome biogenesis was investigated in MP and MA- cells by measuring the amount of [3H] uridine incorporated during 1 h into i) the nuclear 45S pre-rRNA, representing the initial unprocessed rRNA transcript synthesized by RNA pol I and therefore reflecting the the overall rate of 45S pre-rRNA (Fig. 2A) and ii) into the final mature rRNA associated with stable cytoplasmic ribosomes representing an integrated view of all the steps of ribosomal production (Fig. 2B).

For this, in a first step, following the 1 h labeling period with [3H] uridine nuclear RNA was purified, resolved on an agarose gel, transfered to a nitrocellulose membrane, the radioactivity was measured with a phosphorImager (Figure 2A). This analysis showed that the amount of radioactivity incorporated into 45S pre-rRNA of MA- was increase of 20% than that incorporated into the corresponding 45S pre-rRNA of MP (p = 0.05). In parallel, after the 1 h labelling period with [3H] uridine cytoplasmic ribosomes were purified using dedicated methods allowing ribosome purification with a high degree of purity, subunits were separated with high salt concentration containing buffers, tRNAs and mRNAs were eliminated using the amino acid analogue puromycin and RNAse treatment and the radioactivity was measured in equivalent amount of purified ribosomes in a liquid scintillator counter (figure 2B). This analysis showed that the amount of radioactivity incorporated in mature cytoplasmic rRNA of MA- cells was 20% (p = 0,0097) greater than that of the mature cytoplasmic rRNA of MP cells. Altogether these data unambiguously showed that human breast cancer progression is associated with an increase of 20% of the ribosome synthesis.

### Reduced Arl2 content is associated with the activation of an alternative pathway of rRNA processing

**Figure 3:**
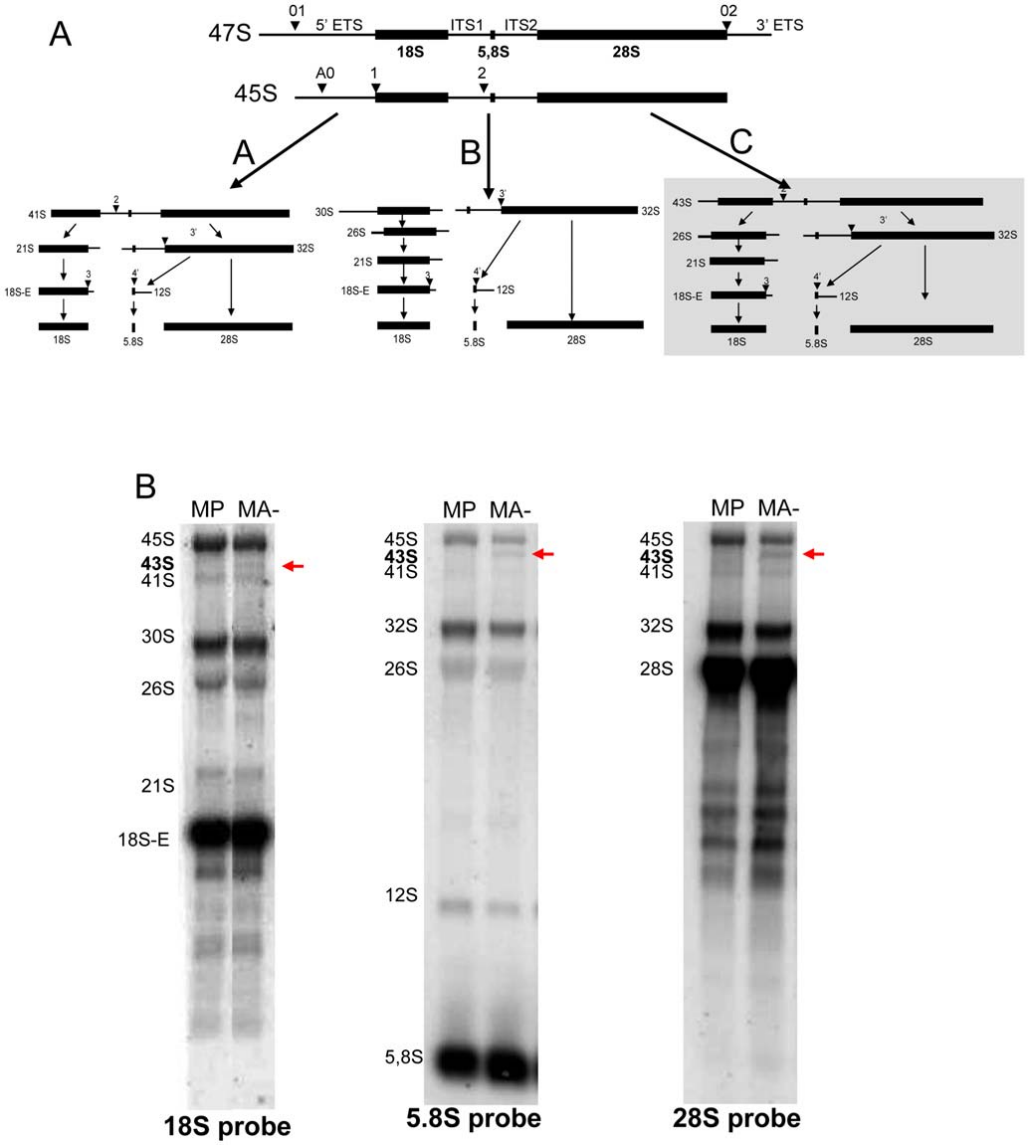
A new rRNA processing pathway is detected in aggressive human breast cancer cells. A: rRNA processing pathways. Simplified diagram of the two major pre-RNA processing pathway in HeLa cells (pathways A and B) and a third chemically-induced pathway (pathway C). Thin lines represent the sequences that are eliminated during the processing (5′ETS, ITS1, ITS2, 3′ETS) to provide mature rRNA (18S, 5.8S and 28S) represented by thick black boxes. Names of the intermediary species refer to the current nomenclature. The positions of the main cleavage sites are indicated by arrowheads and refer to conventional numbering. B: Northern blot analyses. Nuclear RNA (3 µg) was analysed by northern blot using specific probes for the 18S, 5.8S and 28S sequences. Results show that all the pre-rRNA corresponding to the pathways A and B of rRNA maturation exist in MP and MA- cells. A third pathway of rRNA maturation characterized by the presence of the 43S pre-rRNA (red arrow) is evidenced only in the aggressive MA- cell line. This intermediary is specific of the C pathway.

At present, it is generally admitted that there are two different modes of human rRNA post-transcriptional processing [9]. These modes of processing that are schematized in Fig. 3A, designated pathway A and B, involve sequential cleavages of the initial pre-45S rRNA to obtain the matures 18S, 5.8S and 28S. Therefore the first step to analyse the modes of rRNA processing in MP and MA- cells was to design a set of three probes – complementary to the sequences of 18S, 5.8S and 28S - allowing the detection of all the pre-rRNA yet described in human cells. As shown on Fig. 3B, we were able to detect, in both MP and MA- cells, the different subtypes of pre-rRNA that have been previously described in HeLa cells (45S, 41S, 32S, 26S, 18SE, 12S) in addition to the mature rRNA 5.8S and 28S. However, a supplementary 43S intermediate present only in the invasive MA- cells and located between the 45S and the 41S was reproducibly detected, very strongly with the three probes. To verify the specificity of 43S signal detect with the three preliminary probes, we have performed a second northern blot experiment with two additional probes specific of the 5′ETS and 3′ETS. In the case of the 43S pre-rRNA, only the 5′ETS does allow us to detect a signal, whereas 3′ETS does not recognize the pre-rRNA. We have detected a signal in the MA- cell with the 5′ETS probe but no signal with the 3′ETS. No signal was detected with either probe in the control MP cells (data not show). This precursor could be specific of a third rRNA pathway designated pathway C in Fig. 3A.

Our results show that in this model, cancer cells with enhanced aggressivity acquire a supplementary pathway of pre-rRNA processing.

### Methylation pattern of rRNA is modified in cells with reduced Arl2 content

**Figure 4:**
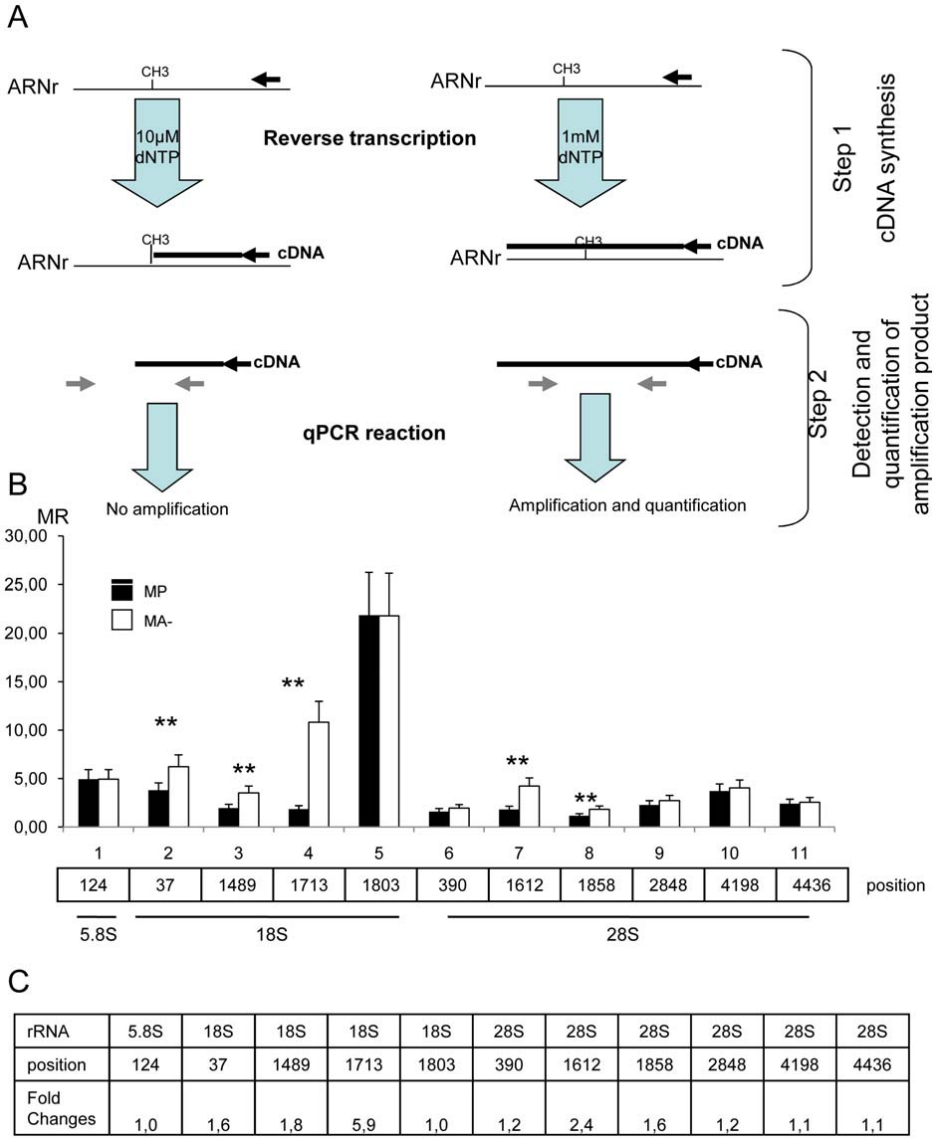
The specificity of the methylation pattern of rRNA is altered in aggressive human breast cancer cells. A: schematic representation of the RT-PCR mapping method used to analyse the methylation pattern of rRNA. The first RT step is identical to the conventional method of rRNA methylations analysis. In the second step however, some adaptations were introduced, first by using a non-radiolabelled probe and next by performing a qPCR of the two RT products. B: Methylation ratio of eleven sites distributed throughout the three rRNA. The position line indicates the number of the nucleotide that is analysed. White bars indicate the methylation ratio (MR) of MP cells and black bars indicate the MR of MA- cells. One site was analysed for the 5.8S, four sites for the 18S and six sites for the 28S according to the method described in the experimental procedure. The MR for the sites analysed on the 5.8S are equivalent in the two cell lines whereas the MR of the sites on the 28S and 18S are higher in MA- cells as compared to control MP cells. Significant differences are indicated by the stars. C: Fold change in the MR between MA- and control MP cells. The fold change represents the calculation of the ratio between the MR of MA- cells and the MR of MP cells. As indicated in this table, the fold changes in the MR between MA- and MP cells are usually between 1.5 and 2 for the selected sites on the 28S rRNA whereas the fold changes are about 1 for selected sites on the 5.8S and 18S rRNA. Only one site (1713) on the 18S rRNA exhibit a fold change in the MR between MA- and MP cells of 6.

We have then analyzed the cytoplasmic rRNA. Extensive northern blot analyzes clearly showed that ribosomes contained only the expected four rRNA species: 28S, 18S, 5.8S and 5S (data not shown). However, because methylation of rRNA is one of its chemical modifications that could play a critical role in its function, the next step of our study was to analyse the methylation pattern of a restricted part of the rRNA of MP and MA- cells. At present the method of reference for this kind of analysis [16], [28] proceeds following two main steps: i) a reverse transcription (RT) using a radioactive oligonucleotide generally located 50 bases dowstream to the methylation site to be analyzed and performed either with a high nucleotide concentration (1 mM) or with a low nucleotide concentration (10 µM); ii) analysis of the resulting radioactive complementary DNA (cDNA) by separation within denaturing polyacrylamide gels and detection by autoradiography or PhosphorImager. The principle of the method is: i) that the eventual methyl group located at the 2′ position of the ribose stops the cDNA synthesis at that place when a low concentration of nucleotide is used whereas cDNA synthesis is not altered when high concentrations of dNTP are used and ii) that separation and relative quantification of radioactive cDNA produced at low and high dNTP concentration allows to determine the presence or the absence of the methyl group. This method is very specific and has been extensively validated. However, it is time consuming, requires the manipulation of radioactive compounds, suffers from a lack of sensitivity, does not allow simultaneous analysis nor more importantly quantification and does not allow us to determine the percentage of multiple rRNA molecules that are methylated at a given position. Therefore to circumvent these limitations, we have modified the existing method. The synopsis of the modified method is presented in Fig. 4A. The first step is very similar to the classical method except that the oligonuceotides used are non-radioactive and that the efficiency of the RT is precisely controlled (see Materials and Methods). The second step is totally different to that of the classical method because the cDNA resulting from both RT (performed with low and high dNTP concentration) are precisely quantified using a qPCR technology described in great details elsewhere[29], [30]. The number of cDNA copies obtained after RT at high concentration is divided by the number of cDNA copies obtained after RT at low concentration. This methylation ratio (MR) gives an evaluation of the percentage of rRNA that is methylated at a given position Using this modified method we have compared the MR between MP and MA- cells from 11 different methylation sites distributed throughout the 5.8S, the 18S and the 28S (figure 4B). For this, the three cytoplasmic rRNA from both cell lines were separated onto an agarose gel, extracted from the gel and submitted to methylation analysis according to the method described in Fig. 4. This analysis showed that the MR of the 28S from MA- were always higher than those of MP cells whatever the sites analyzed suggesting that MA- 28S rRNA and 18S rRNA is more methylated than MP 28S rRNA. Conversely MR of 5.8S of MP and MA- rRNA are very similar suggesting that the level of methylation of these rRNA positions is equivalent in both cell lines. The ratio between the MR of the two cell line are presented in figure 4C, and we can show that this ratio is between 1.4 and 6 fold higher for the most aggressive cell. We have then performed a validation of our novel detection method of rRNA methylation. For three different sites (position 1489 in the 18S rRNA and positions 1856 and 4436 in the 28S rRNA), we have analyzed the methylation ratio according to the standard method. This analysis showed that the methylation ratio measured with either the standard method or the new RT-qPCR method were very similar. Respective fold changes of the rate of methylation between MA- and MP cells for the three sites analyzed were 1.8, 1.6 and 1.1 with the RT-qPCR method and 1.35, 1.85 and 1.06 with the standard method.These results confirmed that the rate of rRNA methylation of at least some sites is higher in the most aggressive cells.

### Reduced Arl2 content is not associated with the protein composition of cytoplasmic ribosomes

**Figure 5:**
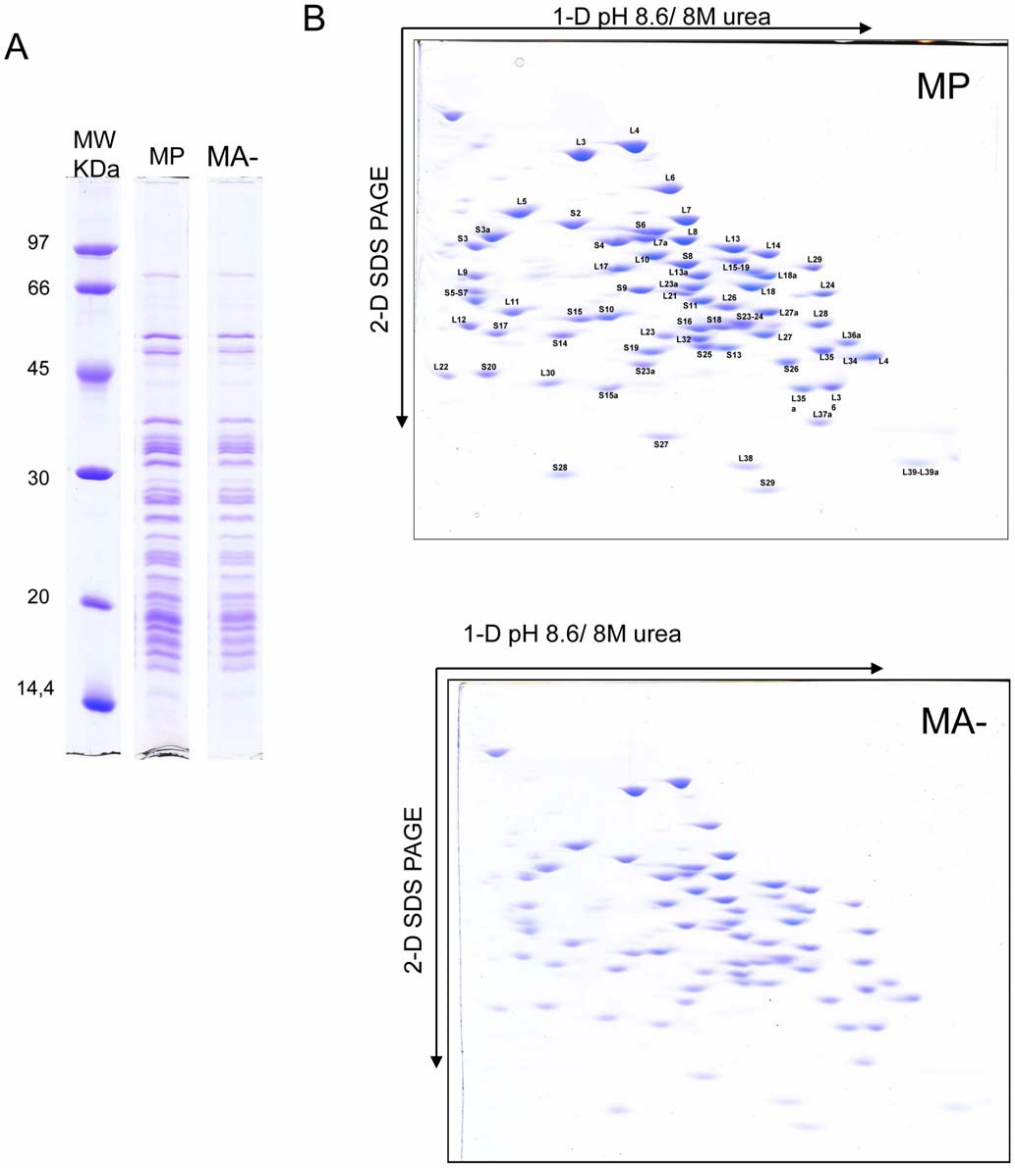
The protein composition of the ribosome is not modified in aggressive human breast cancer cells. The composition of the ribosome from MA- and MP cells was analysed on purified fractions of cytoplasmic ribosome obtained as described in material and methods. Ribosomal proteins were first resolved by 1D SDS-PAGE (panel A), and every ribosomal protein could be clearly detected in the two cell lines, with no apparent quantitative alteration. To increase the resolution of the analysis, ribosomal proteins were next resolved by using a specific 2D-gel system (B) and each protein spot was quantified by image analysis using the ImageMaster© software. Again, no difference was observed in the composition of ribosomal proteins between the MA- and MP cell lines.

To determine more extensively the structure of the ribosome, we have analyzed the composition of the ribosomal protein of the cytoplasmic ribosome of each cell line. After purification of the cytoplamic ribosomes, the ribosomal proteins were extracted and separated by 1D SDS-PAGE (figure 5A). As expected we detect the 80 ribosomal proteins in the two cell lines and the composition seems equivalent in the most aggressive and in the control cells. However, mono-dimensional separation of protein does not permit a fine analysis of the protein composition of the ribosome. We have in a next time performed a two dimensional analysis of ribosomal proteins in a dedicated 2D electrophoresis system that allows to visualize the 80 proteins and to characterize the specific post translational modification of the proteins. Gels are presented in figure 5B. Annotation of each protein is performed with the previous analysis in HeLa cells. All the proteins are detected in the right place and the protein composition of ribosome are similar in the two cell lines. To verify the abundance of each protein, we have performed a bio-informatic analysis using ImageMaster software with 5 gels of each cell line. The signal obtained for each protein are indentical in MP ad MA- cells (figure S1).

Our results show that during human breast cancer progression, invasive cancer cells displays the same composition of ribosomal protein in cytoplasmic ribosome.

### Reduced Arl2 content is associated with dysregulation of translational efficiency

It was recently describe that alteration on ribosome biogenesis is associated with dysregulation of translational efficiency. In particular, ribosome alterations are associated with modification of initiation of translation by IRES element and strong defect in translation fidelity.

**Figure 6:**
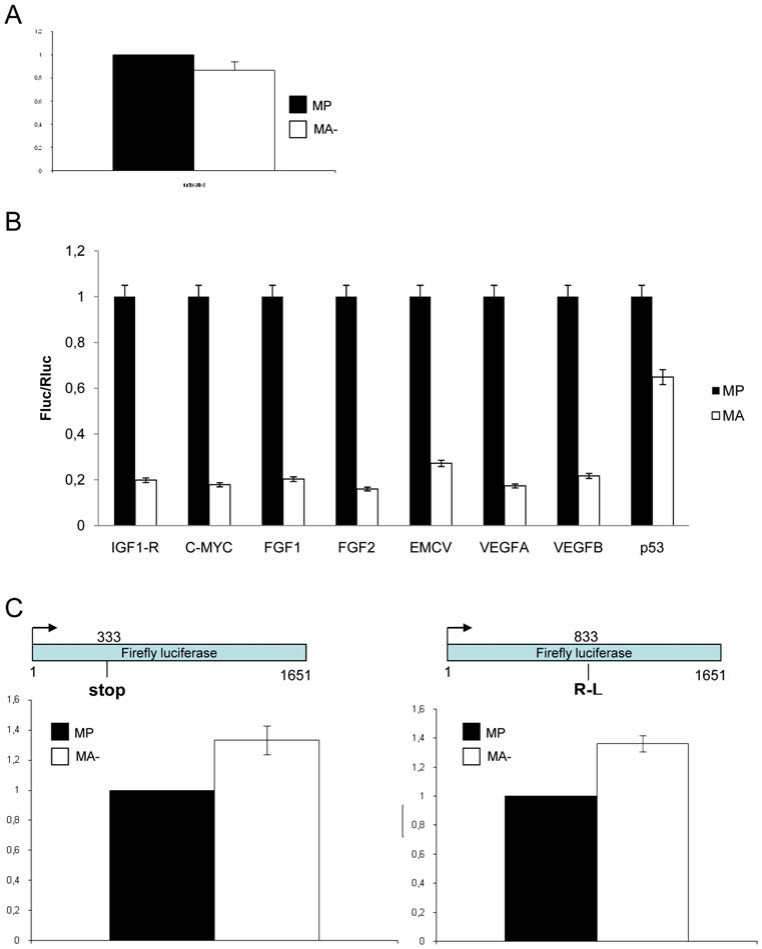
The translational capacity of the ribosome is modified in aggressive human breast cancer cells. A: Rate of global protein synthesis. Cells were incubated in a mixture of ^35^S labelled-cystein and methionine. Total proteins (15 µg) were separated by monodimensional electrophpresis and transferred to a nitrocellulose membrane. The radioactivity incorporated into proteins was measured using a PhosphorImager (Typhoon Trio, GE©). The results represent three independent experiments and the radioactive counting was done in triplicate. In each experimental condition the data obtained (Mean +/− S.D) are expressed as the percentage of those obtained in the control MP cell line. The results show no significant differences in the global rate of protein synthesis between the MA- and the MP cell lines. B: Determination of the translation initiation through IRES. Both *Firefly* and *Renilla Luciferase* were transfected into the cells and activities were measured as described under “materials and methods.” The result for the Firefly luciferase was normalized by the data for Renilla luciferase activity. In each condition of transfection, the data obtained (Mean +/− S.D, n = 5) are expressed as the percentage of those obtained in the control MP cell line. The results clearly indicate that translation initiation through IRES is impaired in MA- cells as compared to MP cells. C: Analysis of translational fidelity in MA- and MP cells. Both cell lines were transfected either with the pGL3m1 vector (stop mutant) or the pGL3 wild type vector. Luciferase activities of the mutant vector and of the pGL3 vector were assessed by co-transfecting the pRL vector. The results of the Firefly luciferase activity of the mutant were normalized by the Firefly luciferase activity of the wild type vector. The data (Mean +/− S.D, n = 5) are expressed as the percentage of those obtained in the control MP cell line. The results indicate that firefly luciferase activity of the mutant vector is highly expressed in MA- cells as compared to MP cells. D: Analysis of translational fidelity in MA- and MP cells using another mutant vector (pGL3m2). Cells were transfected with either the pGL3m2 vector or the pGL3 wild type vector. Luciferase activities of the mutant vector and of the pGL3 vector were assessed by co-transfecting the pRL vector The results of the Firefly luciferase activity of the mutant were normalized by the Firefly luciferase activity of the wild type vector. The data (Mean +/− S.D, n = 5) are expressed as the percentage of those obtained in the control MP cell line. Once again, the results indicate that firefly luciferase activity of the mutant vector is highly expressed in MA- cells as compared to MP cells, clearly demonstrating a strong defect in translational fidelity of MA- cells.

We first analyzed the rate of global protein synthesis. For this, proteins were labelled for 1h by incubation of the cells in a medium containing a mixture of [35S]-methionine and [35S]-cysteine Cells were solubilized and equal amounts of total proteins were separated by SDS-PAGE. Proteins were stained by Coomassie brillant blue, the gels were dried and the radioactivity incorporated in the separated proteins was evaluated using the ImageQuant TL software after PhosphoImager scanning (Figure 6A). No significant difference was observed between MA- and MP suggesting that the global translational activity is equivalent in both cell lines.

We then determined whether the mode of translational initiation, cap- or IRES-dependant was equivalent or different in these cells. For this we used a bi-cistronic vector strategy in which translation of the first cistron is mediated by a cap-dependent scanning mechanism and the second by an IRES-dependant mechanism. These vectors were transfected into MP and MA- cells and translation efficiency of the two coding sequences was evaluated by measuring the two luciferase enzyme activities (Figure 6B). The results were normalized using the Rluc activities. This clearly demonstrated that aggressive MA- cells displayed a significantly lower IRES-dependent translational activity (4-fold less, p<0,05) than that of MP cells whatever the origin of the IRES, either human (p53, IGF-IR, c-myc, FGF1, FGF2, VEGFA and VEGFB) or viral (EMCV). Moreover, usage of an hairpin containing vector (pHBic-943) clearly showed the specificity of the IRES-dependant initiation and allowed to demonstrate that translation of the second cistron was not performed via a terminaison re-initiation mechanisms (Fig. 6B).

We have also analysed two different aspects of the fidelity of translation: nonsense suppression and aa-tRNA selection. To evaluate nonsense suppression we produced a pGL3 firefly luciferase vector containing a premature stop codon. As shown in Fig. 6C, ribosomes of both cell lines displayed the ability to pass through the premature stop codon although this dysfunctional property was 30% higher in the most aggressive cells MA- cells than the control cells MP. To evaluate the aa-tRNA selection we produced a vector in witch the AGA codon was transformed into the near-cognate AGC leucine codon. The R218 residue is a critical amino acid for Fluc activity and this mutation induces the synthesis of a protein with a strongly reduced luciferase activity [14] . Therefore, measuring the level of Fluc activity from this R218A vector allowed us to evaluate the rate of amino acid misincorporation at this position – resulting from altered aa-tRNA selection - since this level reflects the amount of “reverted proteins” in which an R residue was incorporated instead of the expected A residue (Fig. 6C). As expected the level of Fluc activity was low in both cell lines. However, the Fluc activity was 40% higher in MA- cells. This result show that ribosome of aggressive cell lines are deficiency the codon- anticodon recognition.

Our results show that in this model of enhanced tumor aggressivity, the translational capacity of the ribosome of invasive cancer cells is strongly impaired. IRES-initiated translation was less efficient and fidelity of translation was reduced in cells with increased aggressivity.

## Discussion

It is now clearly established that control of ribosome biogenesis is profoundly altered by several oncogenes and/or tumor suppressor [20]. In particular, many studies have demonstrated that over expression of oncogenes or silencing of tumor suppressor induce an increase of RNA *pol* I activity [21], [23], [31]. The data presented in this study show that these alterations are not restricted : i) to only the transcriptional step of ribosomal biogenesis and ii) to the early steps of tumor formation but that important modifications of this fundamental biological process also take place at later stages of tumor evolution, i.e. during the acquisition of the aggressive tumor phenotype. Indeed we demonstrate that breast tumor cells exhibiting an exacerbated aggressive phenotype (MA-) both *in vitro* and *in vivo* display an increase of their rate of ribosome biogenesis accompanied by a modification of the post-transcriptional rRNA processing together with a modification of their translational control ability.

The rate of ribosome biogenesis was evaluated directly by measuring the level of incorporation of ^3^H-uridine within cytoplasmic ribosomes of MP and MA- cells. This unambiguously showed that the rate of ribosome synthesis is higher in the most aggressive cell line, MA-. This result was indeed a confirmation of the observation that the mean size of MA- nucleoli was about 30% higher than that of nucleoli from MP cells and that a significant percentage of MA- cells display more nucleoli per cell. The higher rate of ribosome biogenesis measured in MA- cells was also reflected at the ultra-structural functional organisation of the nucleoli of MA- cells (analyzed by electron microscopy) in which the number of FC and DFC per nucleoli is higher in MA- cells compare to MP cells. Altogether these results demonstrated for the first time that ribosome biogenesis could be over-activated not only during the early steps of tumor progression but also in correlation with acquisition of the aggressive phenotype. Interestingly this also contributes new experimental data supporting the fact that AgNOR could be considered as a “gold standard” pathological feature for clinicians [32] since it could reflect not only the transformed state of a cell but also its level of aggressivity.

Because ribosome biogenesis is a complex multistep process and because the post-transcriptional modifications of the rRNA are crucial for ribosomal functions, the next step of the study was to determine whether these steps of ribosome biogenesis were also modified in the most aggressive cells (MA-) compared to the less aggressive cells (MP). For this we have first demonstrated that the MP cells exhibit the same nuclear pre-rRNA intermediates than those already characterized in another transformed human cell line, the HeLa cell line suggesting that processing of rRNA is very similar in these two cell lines. This processing occurs through two different modes representing different kinetics and different orders of pre-rRNA processing that are conventionally referred to as pathways A and B [9], [10]. Unexpectidely, in MA- cells, a supplementary intermediate was observed demonstrating that a third mode of pre-rRNA processing occurs in this cell line. The existence of a third pre-rRNA processing pathway could have two different potential consequences on the global control of ribosome biogenesis. First, it can be postulated that the post-transcriptional rRNA processing is more efficient in the more aggressive cells (three pathways) compared to the control cells (two pathways) and that this activation of post-transcriptional processing is required, in addition to transcriptional activation, to provide the required amount of cytoplasmic ribosomes. Second, since the post-transcriptional chemical modifications of rRNA occurs during the early steps of processing and since the “abnormal” appearance of 43S pre-rRNA involves a deregulation of the early steps of processing, it can be postulated that the chemical modification profiles of the aggressive cells are different from that of the control cells.

Therefore, because these specific chemical modifications that rRNA undergo during their processing are absolutely crucial for ribosomal function [15], [17]–[19], [33] the next step of our study was to evaluate the ratio of methylation of rRNA. At present, several methods are available to determine the presence of methyl group in rRNA. First, radiolabelling of methyl groups by [^3^H]methyl-methionine incorporation in cell culture. This technique gives only a global vision of the rRNA methylation and does not permit a single point methylation analysis. Moreover, the incorporation of [^3^H]methyl group is dependent of the methylation rate but also of the rate of rRNA synthesis by RNA polymerase I. Second, single methylation point analysis by reverse transcription with radioactive oligonucleotide.

The initial step of the existing method corresponds to two reverse transcription (RT) reactions performed with a specific radioactive oligonucleotide for a target site at high or at low concentration of dNTP. At high concentration, the RT reaction is not blocked by the methyl group whereas at low concentration, the reaction is blocked by the methyl group producing radioactive cDNA of different sizes. The amount of cDNA was then evaluated after electrophoresis and autoradiography. The major limitation of this method is that it does not allow to determine the percentage of rRNA molecule that exhibit a methyl group at a given position. Therefore to circumvent the limitations describe above, we have simply replaced the radioactive detection part of the available method by a well standardized qPCR method (see Results and Experimental procedures sections for methodology). This method allowed us to evaluate the percentage of rRNA molecules methylated at a given position and to determine the methylation ratio (MR) for each selected site. For all the reasons described above we have performed the methylation ratio analysis for 11 sites in the two cell lines distributed in the three main rRNA (5.8S, 18S and 28S) [11] .

The MR for the sites present in the 5.8S of MP and MA- rRNA are very similar suggesting that the level of methylation of these rRNA positions is equivalent in both cell lines. In contrast, the MR of the six sites present in the 28S rRNA and in the 18S rRNA is more elevated in the aggressive MA- cell line than in the control MP cells. As an example, the methylation at position 1858 is two fold higher in the aggressive cells and the methylation at position 1803 is six fold higher. Therefore, the fact that the methylation pattern of mainly the 28S and 18S rRNA seems to be altered suggests that a modulation of ribosomal function could be performed via this mechanism since the peptide bound formation and, to a lesser extent, the tRNA selection activities of the ribosomes are provided by the 28S rRNA and the methylation sites of this rRNA are heavily concentrated in its active sites. However, at this stage the methylation rRNA analysis encompass only 10 % of the total rRNA methylation, therefore we can not exclude that the MR of other sites in the 5.8S and in the 18S vary according to the cell line. The exact function of the methyl groups in the rRNA is not yet clearly understood although it is now well demonstrated that collectively they can control, at least in part, the translational activity of the ribosomes [15], [16], [33]. In the future development of high throughput methods to analyse the level of RNA methylation would be extremely useful.

It has recently been demonstrated that incorrect methylation of rRNA is associated with impaired capacity to initiate translation through IRES and also with a strong defect in translational fidelity [25]. In our study we have demonstrated that the capacity to initiate cap-independent translation by eight different IRES (viral IRES of EMCV and several cellular IRES) is lower in the aggressive MA- cell line and that the ribosome of these cells exhibit a strong deficiency in reading the mRNA. These results suggest that alterations of rRNA maturation observed in aggressive cells have a direct effect on the translational capacity of the ribosome. The modulation of IRES-translation initiation plays a central role in tumorigenesis since IRES elements have been found in several cellular mRNAs including those of different growth factors (for review [34]), proto-oncogenes such as c-*myc* [*35*] transcription factors [36] , and proteins involved in cell cycle progression [37], [38] or apoptosis [39].

Finally, the modification of ribosome biogenesis observed in aggressive MA- cells with reduced Arl2 levels can be explained by the role of p53, since it has been shown that reducing the level of Arl2, suppresses p53 expression [26], [27]and p53 inactivation is associated with up-regulation of RNA polymerase I activity. It now remains to determine whether p53 could modify the post-transcriptional steps of ribosome biogenesis, in particular the level of rRNA methylation

Altogether our results show that breast cancer cells with enhanced aggressivity display profound alterations in ribosome biogenesis resulting in the modification of translational initiation ability of the ribosomes. Further investigations will aim to determine how these alterations, and most notably reduced translational fidelity of these altered ribosomes, are involved in increased tumor aggressivity.

## Materials and Methods

### Cell culture

MP and MA- cell lines were obtained with MCF-7 cells stably transfected with empty pcDNA3 (MP cells) or pcDNA3 containing antisense Arl2 (MA- cells) [27]. The two cell lines were grown in DMEM containing L-glutamine, penicillin (200 IU/ml), streptomycin (200 µg/ml), and foetal bovine serum (10%) at 37°C in the presence of 5% CO2.

### Plasmid construction

Bicistronic plasmid construction and dual luciferase assays have been described [40]. Briefly, the plasmids used were constructed by cloning the RLuc gene as the first cistron under the control of the cytomegalovirus promotor (CAP dependent translation) and the FLuc gene as the second cistron under the influence of IRES of the IGF-IR mRNA (pBiC-943), IRES of p53 or of that coding for the encephalomyocarditis virus (EMCV) IRES (pBiCV). The pHBIC-943 vector is identical to the pBIC-943 vector except for the introduction of a hairpin sequence before the RLuc gene. Vector containing IRES of c-myc, FGF1, FGF2, VEGFA and VEGFB are provided by the group of Anne Catherine Prats [41] . pGL3 mutant stop vector (pGL3m1) corresponds to the Firefly luciferase of the pGL3 vector with a premature stop codon (UAG) at the position 333 of the coding sequence. pGL3 misincorporation test reporters (pGL3m2) was created by mutating the firefly luciferase catalytic residue R218 from the wild-type AGA codon to the near-cognate AGC leucine codon of the pGL3 vector. Mutant sequences were obtained by site-directed mutagenesis using PCR from 30 ng of pGL3 vector (Promega), pfu DNA polymerase (Stratagene) and the following primers (Eurogentec):

333-5′-GGAAGATGGAACCGCTGGATAGCAACTGCATAAGG-3′

333-5′-CCTTATGCAGTTGCTATCCAGCGGTTCCATCTTCC-3′.

R218-5′-CTAAGGAAGTCGGGGUAGCGGTTGCCAAGAGGTTC-3′

R218-5′-GAACCTCTTGGCAACCGCACCCCGACTTCCTTAG-3′

In order to standardize the pGL3 mutants and the pGL3 wild type expression, we performed a co-transfection with the pRL vector coding for renilla luciferase Transfection were made at the ratio ¼–¾ for mutant vector and pRL respectively. These plasmids were transfected into MP, and MA- cells using lipofectamine 2000 (Invitrogen) according to the manufacturer information and tested with dual luciferase (Promega) ..

### Fluorescence and electron microscopy

Cells were processed for electron microscopy as previously described [22]. For immunofluorescence analysis, cells were grown on glass coverslips, fixed with 4 % of paraformaldehyde in PBS before permeabilization with 1 % Triton X-100 in Phosphate Buffered Saline (PBS). B23 and nucleolin proteins were localized using respectively mouse monoclonal antibodies B0556 (Sigma) and rabbit polyclonal antibody (P. Bouvet, ENS Lyon) and a FluoProbes 546 anti-mouse secondary antibody (Interchim). Coverslips were mounted using the Fluoromount G medium. DNA staining was performed using di-aminido-phenyl-indol (DAPI) (Roche, Manheim, Germany) according to manufacturer instructions. Staining was visualized at room temperature by Fluorescent microscopy. Images were captured using an AxioCam HR camera, acquired with the LSM software (Zeiss). Signal quantification was performed with Image J software (USA).

### RT-qPCR analysis of the methylation pattern of rRNA

Methylation of rRNA was analyzed by RT-qPCR. A total of 11 methylation sites was selected for 5.8S, 18S and 28S. Reverse transcription of rRNA into cDNA was realized using 100 ng of purified 5.8S, 18S and 28S rRNA, extracted from MA- and MP cells, in the presence of 200 units of M-MLV reverse transcriptase, RNase H minus (Promega M3683), 40 units of RNasin Ribonuclease inhibitor (Promega N2515), 1 µM of each reverse primer targeting a sequence upstream to a specific methylation site, and either 10 µM or 1 mM dNTPs. In each reaction tube, 7.5×10^8^ copies of a synthetic polyA RNA (SmRNA), used as an external standard, were added to calibrate the efficiency of reverse transcription (Morales and Bezin, patent WO2004.092414), as previously described (Navarro et al., 2008). Reverse transcription has been performed at 42°C for 90 min, and then stopped by an incubation at 70°C during 15 min. Quantitative amplification of the targeted cDNAs was performed by PCR using a Lightcycler 1.2 (Roche Diagnostics), and the QuantiTect SYBR Green PCR kit (Qiagen). Sequences of the primers used for PCR are provided (table S1 )

### Rate of protein synthesis

Cells were incubated one hour in methionine-free cysteine-free DMEM (Gibco) supplemented with 10% inactivated and dialyzed FCS and a mix of [35S] methionine and [35S] cysteine at final concentrations of 75 µCi/ml (GE healthcare). After 1 h of labelling, medium was removed, cells were washed three times with ice cold PBS. Cells were disrupted in Laemmli buffer and an aliquot of each sample containing an equal amount of proteins was separated in a 12.5% polyacrylamide gel. Gels were stained with Coomassie blue, vacuum dried and analyzed by phosphoImager (typhoon trio, GE Healthcare).

### Rate of polymerase I activity and of ribosome synthesis

Cells were incubated one hour in DMEM supplemented with 10% inactivated FCS and [5,6–3H] uridine at a final concentration of 15 µCi/ml (GE healthcare). At the end of labelling time, cells were washed three times with ice cold PBS containing 10–4M of uridine. For RNA polymerase I activity, radioactive nuclear RNAs were separated in formaldehyde agarose gels, transferred to nitrocellulose membranes. Radioactivity of the 45S pre-rRNA was measured after exposure of the membranes using a PhosphorImager system (Typhoon and ImageQuant TL, Amersham). To determine the rate of ribosome synthesis a post mitochondrial fraction was prepared as previously described [42]. This supernatant was treated for 15 min at 0°C followed by 10 min at 37°C with puromycin at a final concentration of 10–4M and then adjusted to 100 mM CaCl2 before treatment with 100 Units per ml of micrococcal nuclease (Boerhinger) for 15 min at 20°C. Ribosomes were purified by centrifugation through a 1M sucrose cushion containing 0,5M KCl and quantified. An amount of ribosomes corresponding to one A260 unit was mixed with 10 ml scintillator (Beckman) and the amount of radioactivity was measured in a liquid scintillation counters (Perkin).

### 2D analysis of ribosomal proteins

Ribosomes and ribosomal proteins were purified as previously described in detail [43]–[45]. After purification proteins were alkylated with iodoacetamide and separated by two dimensional 2D-PAGE as described in details elsewhere [46]. In brief, 2.5 OD260 units of each ribosomal protein preparation were separated in the first dimension in a 4% (w/v) polyacrylamide gel containing 8M urea at pH 8.6. The second dimension separation was performed in a 12.5% polyacrylamide gel containing 6M urea at pH 6.75 in the presence of SDS. Gels were stained with Coomassie brillant blue R250. The intensity of each spot was determined with the software Image Master (GE Healthcare).

### Northern blot analysis

Nuclear fraction was obtained after cell disruption 5 min at 0°C using buffer RLN (50 mM Tris-HCl pH 8, 140 mM NaCl, 1,5 mM MgCl2 et 0,5% (v/v) Nonidet P-40). Nuclear RNA was purified using the RNeasy kit (Qiagen) and quantified with a nanodrop spectrophotometer (Thermo-Scientific). Nuclear RNA were separated in formaldehyde agarose gels, transferred to nitrocellulose membranes. Sequences of the oligonucleotide probes (Invitrogen) used for northern blot analyses are the following: 18S- 5′-ATCGGCCCGAGGTTATCTAGAGTCACCAAA-3′, 28S- 5′-CCTCTTCGGGGGACGCGCGCGTGGCCCCGA-3′ and 5.8S- 5′-TCAGACAGGCGTAGCCCCGGGAGGAACCCG-3′. For labeling, 50 pmoles of each probe were incubated with 50 pmoles of [?-32P] ATP and T4 polynucleotide kinase (Promega) during 30 min at 37°C.Northern blot hybridization was performed as described in [47] and signal detection was performed by PhosphorImager (Typhoon, GE Healthcare) scanning.

## Supporting Information

Figure S1 Signal quantification of ribosomal proteins. Five gels of ribosomal protein stained with Coomassie blue of the two cell lines were analyzes using the ImageMaster software. Spot were detected and the intensity of each spot were quantified. Annotation of spot were performed using ribosome cartography made in HeLa cells. The intensity of signal corresponding of the proteins of the large subunit are presented in panel A and the intensity of ribosomal protein of the small subunit are presented in panel B. For all the ribosomal protein, the signal intensity of each ribosomal proteins is equivalent in the two cell lines.
(0.10 MB TIF)

Table S1 Oliginucleotides for detection of methylation by RT-qPCR and amplification product size.
(0.04 MB DOC)
