# Supplementary figures and images for: Dysregulation of Ribosome Biogenesis and Translational Capacity Is Associated with Tumor Progression of Human Breast Cancer Cells
Source: PLoS One. 2009 Sep 25;4(9):e7147. doi: 10.1371/journal.pone.0007147 (PMC2744998; doi:10.1371/journal.pone.0007147)

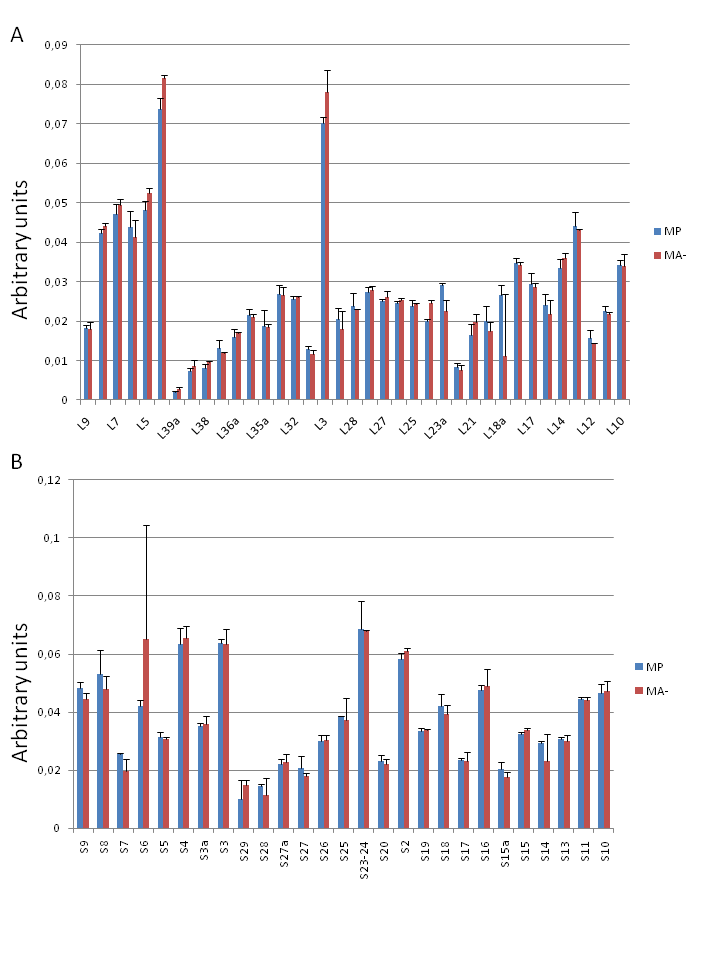

Supplement: Figure S1 — Signal quantification of ribosomal proteins. Five gels of ribosomal protein stained with Coomassie blue of the two cell lines were analyzes using the ImageMaster software. Spot were detected and the intensity of each spot were quantified. Annotation of spot were performed using ribosome cartography made in HeLa cells. The intensity of signal corresponding of the proteins of the large subunit are presented in panel A and the intensity of ribosomal protein of the small subunit are presented in panel B. For all the ribosomal protein, the signal intensity of each ribosomal proteins is equivalent in the two cell lines. (0.10 MB TIF) [file pone.0007147.s001.tif]
