# Supplementary material for: Dysregulation of Ribosome Biogenesis and Translational Capacity Is Associated with Tumor Progression of Human Breast Cancer Cells
Source: PLoS One. 2009 Sep 25;4(9):e7147. doi: 10.1371/journal.pone.0007147 (PMC2744998; doi:10.1371/journal.pone.0007147)
Supplement: Table S1 — Oliginucleotides for detection of methylation by RT-qPCR and amplification product size. (0.04 MB DOC) [file pone.0007147.s002.doc]

| oligonucleotide | Sequence 5’-3’ | Product size |
| --- | --- | --- |
| 5.8S-124 left | AGCTGCGAGAATTAATGTGAA | 60 |
| 5.8S-124 right | AAGTGCGTTCGAAGTGTCG |  |
| 18S-37 left | ACCTGGTTGATCCTGCCAGT | 72 |
| 18S-37 right | GGCCGTGCGTACTTAGACAT |  |
| 18S-1489 left | CACCCGAGATTGAGCAATAA | 81 |
| 18S-1489 right | CGCTGAGCCAGTCAGTGTAG |  |
| 18S-1713 left | GTCCCTGCCCTTGTACACAC | 54 |
| 18S-1713 right | CACTAAACCATCCAATCGGTA |  |
| 18S-1803 left | GCGGAGCGCTGAGAAGAC | 89 |
| 18S-1803 right | GATCCTTCCGCAGGTTCAC |  |
| 28S-390 left | CCGTAAGGGAAAGTTGAAAAG | 78 |
| 28S-390 right | CCCCACCCGTTTACCTCTTA |  |
| 28S-1612 left | CGGTCCTGACGTGCAAAT | 84 |
| 28S-1612 right | TCGGAGGGAACCAGCTACTA |  |
| 28S-1858 left | GTGGGCCACTTGGTAAGC | 91 |
| 28S-1858 right | TTTCTGGGGTCTGATGAGC |  |
| 28S-2848 left | AGGTAAGGGAAGTCGGCAAG | 62 |
| 28S-2848 right | CAGCCCTTAGAGCCAATCCT |  |
| 28S-4198 left | GCGGTACACCTGTCAAACG | 69 |
| 28S-4198 right | CACGGGAGGTTTCTGTCCT |  |
| 28S-4436 left | CGCTTTTTGACCTTCGATGT | 51 |
| 28S-4436 right | GCGAATTCTGCTTCCAATGA |  |

table S1 : oliginucleotides for detection of methylation by RT-qPCR and amplification product size.
